# Use and misuse of the self-control concept in the public sphere

**DOI:** 10.3389/fpsyg.2024.1457524

**Published:** 2024-08-30

**Authors:** Michail D. Kokkoris

**Affiliations:** School of Business and Economics, Department of Marketing, Vrije Universiteit Amsterdam, Amsterdam, Netherlands

**Keywords:** self-control, stigmatization, vulnerable groups, social injustice, reductionism, paternalism, phenomenology, nudges

## Introduction

While self-control has become a major focus in social psychology, its influence extends well-beyond academia: self-help books, motivational speakers, and wellness influencers all promote self-control as a solution to many life problems and as a valuable trait everyone should strive to cultivate. The glorification of self-control in public discourse and popular media underscores the need for a deeper examination of its societal impact. This article critically examines five common misinterpretations and misapplications of the self-control concept in the public sphere. By placing self-control within a broader societal context, I argue that the tendency to use insights from self-control research to explain complex social problems might obscure critical determinants of social issues, harm vulnerable groups and individuals, and steer policy preferences in directions favoring individual accountability over collective solutions to social problems.

## Misalignment of scientific and lay conceptions

A first issue with the lay understanding of the self-control concept concerns a discrepancy between how self-control actually works and how people believe it works. This discrepancy can be quite consequential because lay theories of self-control influence actual behavior (Freeman et al., 2013; Job et al., 2010, 2013; Mukhopadhyay and Johar, 2005). Research has shown that effective self-control does not always require effortful inhibition (Fujita, 2011). Instead, smart strategies that help individuals stay on track (e.g., by minimizing exposure to temptations in the first place) seem to be equally or even more effective (De Ridder, 2024; Duckworth et al., 2018; Milyavskaya et al., 2021).

However, lay people might not share this perspective. Their conception of self-control is more in line with a “no pain, no gain” view (Gennara et al., 2023). What people recognize as successful self-control is a combination of effortful processes and positive outcomes. For example, people believe they have made more progress toward a goal (i.e., exercising), if the alternatives they had to resist were more rather than less tempting (Rafieian and Sharif, 2023). Similarly, individuals feel more proud of themselves when making a healthy food choice requires resisting more tempting unhealthy options compared to less tempting ones (Dhar and Wertenbroch, 2012). From an observers' perspective, for actions requiring equal effort, people are more likely to view an action as requiring self-control when the outcome is moral or socially desirable (Newman et al., 2015). This means that outcome valence influences observers' perceptions, who seem to overlook the possibility that self-control may also be necessary to achieve negative or socially undesirable outcomes (Kokkoris and Stavrova, 2020; Kopetz and Orehek, 2015; Rawn and Vohs, 2011).

Overall, people tend to view self-control through a more ascetic, onerous or “Protestant” lens than what current research intends with this term. This implies that the widespread media buzz about self-control might be mistakenly interpreted by lay people as an encouragement to sacrifice momentary enjoyment and forego pleasure for a successful life, which does not necessarily lead to the desired outcomes one envisions (Inzlicht and Roberts, 2024). In fact, goal pursuit can benefit from including planned hedonic deviations in the goal-striving plan (do Vale et al., 2016), from focusing on moderation rather than abstinence (Le et al., 2024), and from viewing pleasure as part of the solution to self-control problems rather than as the problem itself (Becker and Bernecker, 2023).

## Stigmatization of vulnerable populations

Demonizing the lack of self-control can stigmatize and harm already vulnerable populations. Take obesity as an example, which is a classic case of an issue for which personal responsibility remains the dominant explanation in public discourse, despite a wealth of evidence showing that obesity stems from a wide range of genetic and environmental factors most of which are outside an individual's control (Westbury et al., 2023). Attributing visible traits like higher body weight to a lack of self-control can induce negative perceptions and moral judgments. By association, it can lead to all the negative inferences of low self-control—such as being untrustworthy (Righetti and Finkenauer, 2011), antisocial (Fitouchi et al., 2023), immoral (Mooijman et al., 2018), or even subtly dehumanized by being mentally associated with unrefined animals (Haslam et al., 2007)—being projected onto individuals in already disadvantageous conditions, which might have little to do with a lack of self-control, such as obesity, poverty, or substance abuse.

Stigma can also be internalized and negatively impact the lives of disadvantaged groups. For example, the perpetuation of obesity stigma has severe consequences for the mental health of overweight and obese individuals (Alimoradi et al., 2020; Pereira-Miranda et al., 2017; Papadopoulos and Brennan, 2015). A recent large meta-analysis found a negative impact of perceived obesity stigma on mental health over and above the impact of obesity *per se* (Emmer et al., 2020). Obesity stigma also harms physical health. Popular beliefs, reflected also in public health policies, suggest that social pressure and making obesity socially unacceptable can encourage people with obesity to lose weight. In contrast, empirical evidence suggests that, ironically, obesity stigma actually increases the risk of obesity (Westbury et al., 2023). Additionally, obesity stigma is associated with an increased mortality risk of almost 60%, even after controlling for common risk factors, including BMI (Sutin et al., 2015).

While highlighting the benefits of self-control might seem like a noble goal, inferring negative attributes from its lack or reducing complex issues to self-control deficiencies can be harmful. This approach projects negative traits onto individuals, increasing prejudice and causing them to internalize these perceptions with adverse implications.

## Perpetuation of social injustice

Appealing to self-control failures can foster victim-blaming, which is defined as the tendency to hold individuals fully or partially responsible for their misfortunes (Johnson et al., 2021). Victim blaming not only affects the credibility of individuals but also of a collective as a whole. This tendency is closely linked to just world beliefs, the notion that people get what they deserve, which can decrease people's empathy with those facing hardship, even when it is through no fault of their own (Lerner, 1965; Lerner and Miller, 1978). In turn, this can perpetuate social injustice, by undermining support for necessary policy reforms and collective action aimed at addressing root causes.

Self-control can serve as a basis for outgroup derogation. Many groups typically marginalized in the West, such as non-Westerners, women, children, LGBTQ+ individuals, obese people, those in poverty, or drug users and smokers, might be construed as deficient in self-control and therefore seen as less worthy of respect (Joffe and Staerklé, 2007). Internal attributions of responsibility can also perpetuate inequality. For example, research has found that activating the belief that life outcomes stem from personal responsibility rather than from societal factors increases justification of wealth inequality and decreases support for redistributing educational resources, raising taxes on the rich, and promoting policies for intergroup equality and societal benefits (Savani and Rattan, 2012; Savani et al., 2011). Moreover, experiencing a higher sense of control and power can hinder perspective-taking and lead to harsher judgments of others. For example, when individuals experience a higher sense of personal control they are more likely to hold others more accountable of their actions (Cornwell and Higgins, 2019). Similarly, when people have more power they perceive others as having more choice and are therefore more likely to blame and punish them for poor performance (Yin et al., 2022).

These insights suggest that prioritizing self-control as a dominant principle in public discourses about social issues or approaching these issues from a perspective of heightened control and power can have the consequence that disadvantaged groups are perceived as worthy of their suffering and therefore undeserving of help. This view is not far from regimes of responsibilization that emphasize individual accountability over collective solutions in many spheres of life, including health, finances, and education (Giesler and Veresiu, 2014).

## Reductionism of complex social issues

Lay people and researchers alike often assume that “self-control failures contribute to a range of policy issues, from educational achievement […] and retirement savings […] to the obesity epidemic” (Duckworth et al., 2018, p. 102). Research in this area often references these broader social issues to justify the importance of studying self-control at the individual level, because “low self-control is assumed to be at the heart of many societal problems, including obesity, substance abuse, criminality, impulsive buying, and procrastination” (De Ridder et al., 2012; p. 76). Moreover, “inadequate self-control has been linked to behavioral and impulse-control problems, including overeating, alcohol and drug abuse, crime and violence, overspending, sexually impulsive behavior, unwanted pregnancy, and smoking” (Baumeister et al., 2007; p. 351).

Reducing broader social problems to individual-level shortcomings oversimplifies complex issues, neglecting structural and systemic causes behind them. For instance, whereas the obesity epidemic is frequently cited as a prime example of self-control failure (e.g., Duckworth et al., 2018; p. 102), obesity rates have started rising in the Western world only recently with no evidence of a simultaneous global drop in self-control levels (Loewenstein, 2018). Instead, increasing inequalities during this period might have driven this obesity trend, in line with abundant research showing the significant impact of socioeconomic factors on eating behaviors (Best and Papies, 2019; Drewnowski, 2009; Lee, 2011; Pigeyre et al., 2016). Other explanations of this increase in obesity rates can be economic factors (Pancrazi et al., 2022), lifestyle changes (Silveira et al., 2022), and structural changes in the food industry (Swinburn et al., 2011). The same case can be made for the excessive use of smartphones and social media, which is often attributed to poor self-control (Berger et al., 2018; Kim et al., 2016). However, these behaviors are significantly influenced by the design of digital platforms that heavily invest in user engagement (Alter, 2017; Turel et al., 2014; Zuboff, 2019) or by societal shifts toward digital connectivity and the normalization of constant online presence (Twenge et al., 2019).

Focusing on self-control to explain complex social phenomena might obscure the contribution of more critical determinants and divert policymakers' attention from other factors that may have a higher potential to inspire effective interventions. Whereas this reductionist approach permeates many domains of psychological research, the proliferation and significant societal implications of self-control research (Duckworth et al., 2018) make it particularly imperative to address the limitations of this approach in this domain.

## Paternalistic assumptions in welfare

Another widespread belief is that helping individuals exert self-control is unconditionally beneficial. Based on this premise, behavioral science has focused on designing nudges—minimal, low-effort interventions—to enhance individuals' self-control (Broers et al., 2017; Bucher et al., 2016; Hummel and Maedche, 2019). Examples include encouraging people to take the stairs instead of the elevator or to grab a fruit instead of a chocolate bar at the cafeteria check-out.

However, some nudges may inadvertently harm individuals by adopting a paternalistic view in contexts where the nudger is unable to properly determine individuals' true preferences (Sunstein, 2016). This objection to nudges might be particularly relevant and in need of discussion in the field of self-control. From the point of view of individual and social welfare, self-control is valuable because it is a means to an end (i.e., a vehicle to achieve desirable outcomes). But exerting self-control—and more crucially, *not* exerting self-control—might also have a welfare value in its own right. Research reveals that the subjective value of self-control might vary across individuals or across time. Individuals who rely more on feelings (vs. reason) when making decisions experience self-control as alienating (i.e., as if they are betraying their true selves; Kokkoris et al., 2019). Additionally, for individuals who value the enactment of temptations more, indulging induces less guilt or shame (Ghoniem and Hofmann, 2021). The temporal perspective also plays a role. Individuals regret exerting self-control (vs. indulging) with greater temporal distance from a recalled event because of higher affective (vs. cognitive) processing (Kivetz and Keinan, 2006). These findings highlight the importance of a largely neglected research topic: the subjective experience or the phenomenology of self-control. Additionally, self-control and delay of gratification might not pay off if environmental conditions are unstable or resources are unavailable in the future (McGuire and Kable, 2013; Reynolds and McCrea, 2019). Under these conditions, individuals might not reap the fruits of their sacrifices, rendering self-control a maladaptive decision.

It is therefore crucial—although perhaps uncomfortable—to acknowledge that self-control may not always align with personal values and that indulgence might also have welfare value in some situations. In designing welfare-maximizing interventions, policy makers need to adopt a holistic approach to individual welfare (e.g., Logel et al., 2015) by taking into account not only the normative and instrumental effects of self-control, but also the welfare value of individuals' subjective experiences (Kokkoris, 2024).

## Conclusion

The aim of this article is to raise awareness about the societal impact of self-control research, specifically how the concept is perceived and applied in the public sphere. Academic research can shape people's preferences, policymakers' priorities, and corporations' strategies. A disproportionate emphasis on the individual level in self-control research (Hofmann, 2024) or a misinterpretation of empirical findings can shift responsibilities from political and corporate shoulders to individuals' shoulders (Giesler and Veresiu, 2014), with potentially detrimental consequences for public health (Hook and Markus, 2020) and society at large (Madan et al., 2020). This opinion article serves as a call to action for more systematic and responsible dissemination of research findings on the consequential concept of self-control to the public.

## References

[B1] Alimoradi Z. Golboni F. Griffiths M. D. Broström A. Lin C. Y. Pakpour A. H. (2020). Weight-related stigma and psychological distress: a systematic review and meta-analysis. Clin. Nutr. 39, 2001–2013. 10.1016/j.clnu.2019.10.016 31732288

[B2] Alter A. (2017). Irresistible: The Rise of Addictive Technology and the Business of Keeping Us Hooked. New York, NY: Penguin Press.

[B3] Baumeister R. F. Vohs K. D. Tice D. M. (2007). The strength model of self-control. Curr. Dir. Psychol. Sci. 16, 351–355. 10.1111/j.1467-8721.2007.00534.x

[B4] Becker D. Bernecker K. (2023). The role of hedonic goal pursuit in self-control and self-regulation: Is pleasure the problem or part of the solution? Affect. Sci. 4, 470–474. 10.1007/s42761-023-00193-2 37744979 PMC10514018

[B5] Berger S. Wyss A. M. Knoch D. (2018). Low self-control capacity is associated with immediate responses to smartphone signals. Comput. Human Behav. 86, 45–51. 10.1016/j.chb.2018.04.031

[B6] Best M. Papies E. K. (2019). Lower socioeconomic status is associated with higher intended consumption from oversized portions of unhealthy food. Appetite 140, 255–268. 10.1016/j.appet.2019.05.009 31082447

[B7] Broers V. J. De Breucker C. Van den Broucke S. Luminet O. (2017). A systematic review and meta-analysis of the effectiveness of nudging to increase fruit and vegetable choice. Eur. J. Public Health 27, 912–920. 10.1093/eurpub/ckx085 28655176

[B8] Bucher T. Collins C. Rollo M. E. McCaffrey T. A. De Vlieger N. Van der Bend D.. (2016). Nudging consumers towards healthier choices: a systematic review of positional influences on food choice. Br. J. Nutr. 115, 2252–2263. 10.1017/S0007114516001653 27185414

[B9] Cornwell J. F. Higgins E. T. (2019). Sense of personal control intensifies moral judgments of others' actions. Front. Psychol. 10:2261. 10.3389/fpsyg.2019.02261 31636593 PMC6787679

[B10] De Ridder D. (2024). Getting a grip on yourself or your environment: creating opportunities for strategic self-control in behavioral public policy. Soc. Personal. Psychol. Compass 18:e12952. 10.1111/spc3.1295211239803

[B11] De Ridder D. T. Lensvelt-Mulders G. Finkenauer C. Stok F. M. Baumeister R. F. (2012). Taking stock of self-control: a meta-analysis of how trait self-control relates to a wide range of behaviors. Person. Soc. Psychol. Rev. 16, 76–99. 10.1177/1088868311418749 21878607

[B12] Dhar R. Wertenbroch K. (2012). Self-signaling and the costs and benefits of temptation in consumer choice. J. Market. Res. 49, 15–25. 10.1509/jmr.10.049011670861

[B13] do Vale R. C. Pieters R. Zeelenberg M. (2016). The benefits of behaving badly on occasion: successful regulation by planned hedonic deviations. J. Consum. Psychol. 26, 17–28. 10.1016/j.jcps.2015.05.001

[B14] Drewnowski A. (2009). Obesity, diets, and social inequalities. Nutr. Rev. 67, S36–S39. 10.1111/j.1753-4887.2009.00157.x 19453676

[B15] Duckworth A. L. Milkman K. L. Laibson D. (2018). Beyond willpower: strategies for reducing failures of self-control. Psychol. Sci. Public Int. 19, 102–129. 10.1177/1529100618821893 30760176

[B16] Emmer C. Bosnjak M. Mata J. (2020). The association between weight stigma and mental health: A meta-analysis. Obes. Rev. 21:e12935. 10.1111/obr.12935 31507062

[B17] Fitouchi L. Andr,é J. B. Baumard N. (2023). Moral disciplining: the cognitive and evolutionary foundations of puritanical morality. Behav. Brain Sci. 46:e293. 10.1017/S0140525X22002047 36111617

[B18] Freeman N. Shmueli D. Muraven M. (2013). Lay theories of self-control influence judgments of individuals who have failed at self-control. J. Appl. Soc. Psychol. 43, 1418–1427. 10.1111/jasp.12098

[B19] Fujita K. (2011). On conceptualizing self-control as more than the effortful inhibition of impulses. Person. Soc. Psychol. Rev. 15, 352–366. 10.1177/1088868311411165 21685152

[B20] Gennara A. Peetz J. Milyavskaya M. (2023). When more is less: self-control strategies are seen as less indicative of self-control than just willpower. J. Exp. Soc. Psychol. 106:104457. 10.1016/j.jesp.2023.1044579247847

[B21] Ghoniem A. Hofmann W. (2021). When impulsive behaviours do not equal self-control failures: the (added) value of temptation enactments. Eur. J. Pers. 35, 267–288. 10.1002/per.2280

[B22] Giesler M. Veresiu E. (2014). Creating the responsible consumer: moralistic governance regimes and consumer subjectivity. J. Consum. Res. 41, 840–857. 10.1086/677842

[B23] Haslam N. Loughnan S. Reynolds C. Wilson S. (2007). Dehumanization: a new perspective. Soc. Personal. Psychol. Compass 1, 409–422. 10.1111/j.1751-9004.2007.00030.x

[B24] Hofmann W. (2024). Going beyond the individual level in self-control research. Nat. Rev. Psychol. 3, 56–66. 10.1038/s44159-023-00256-y

[B25] Hook C. J. Markus H. R. (2020). Health in the United States: are appeals to choice and personal responsibility making Americans sick? Perspect. Psychol. Sci. 15, 643–664. 10.1177/1745691619896252 32097096

[B26] Hummel D. Maedche A. (2019). How effective is nudging? A quantitative review on the effect sizes and limits of empirical nudging studies. J. Behav. Exp. Econ. 80, 47–58. 10.1016/j.socec.2019.03.005

[B27] Inzlicht M. Roberts B. W. (2024). The fable of state self-control. Curr. Opin. Psychol. 58:101848. 10.1016/j.copsyc.2024.101848 39096668

[B28] Job V. Dweck C. S. Walton G. M. (2010). Ego depletion—Is it all in your head? Implicit theories about willpower affect self-regulation. Psychol. Sci. 21, 1686–1693. 10.1177/0956797610384745 20876879

[B29] Job V. Walton G. M. Bernecker K. Dweck C. S. (2013). Beliefs about willpower determine the impact of glucose on self-control. Proc. Nat. Acad. Sci. U. S. A. 110, 14837–14842. 10.1073/pnas.1313475110 23959900 PMC3773743

[B30] Joffe H. Staerklé C. (2007). The centrality of the self-control ethos in western aspersions regarding outgroups: a social representational approach to stereotype content. Cult. Psychol. 13, 395–418. 10.1177/1354067X07082750

[B31] Johnson V. E. Nadal K. L. Sissoko D. G. King R. (2021). “It's not in your head”: gaslighting,splaining, victim blaming, and other harmful reactions to microaggressions. Perspect. Psychol. Sci. 16, 1024–1036. 10.1177/17456916211011963 34498522

[B32] Kim Y. Jeong J. E. Cho H. Jung D. J. Kwak M. Rho M. J.. (2016). Personality factors predicting smartphone addiction predisposition: behavioral inhibition and activation systems, impulsivity, and self-control. PLoS ONE 11:e0159788. 10.1371/journal.pone.0159788 27533112 PMC4988723

[B33] Kivetz R. Keinan A. (2006). Repenting hyperopia: an analysis of self-control regrets. J. Cons. Res. 33, 273–282. 10.1086/506308

[B34] Kokkoris M. Stavrova O. (2020). The Dark Side of Self-Control. Harvard Business Review.

[B35] Kokkoris M. D. (2024). Self-control and self-expression. Curr. Opin. Psychol. 58:101846. 10.1016/j.copsyc.2024.101846 39089088

[B36] Kokkoris M. D. Hoelzl E. Alós-Ferrer C. (2019). True to which self? Lay rationalism and decision satisfaction in self-control conflicts. J. Person. Soc. Psychol. 117:417. 10.1037/pspp0000242 30920281

[B37] Kopetz C. Orehek E. (2015). When the end justifies the means: self-defeating behaviors as “rational” and “successful” self-regulation. Curr. Dir. Psychol. Sci. 24, 386–391. 10.1177/0963721415589329

[B38] Le P. Q. Scholer A. A. Fujita K. (2024). The role of conflict representation in abstinence versus moderation in self-control. J. Pers. Soc. Psychol. 126, 947–977. 10.1037/pspa0000381 38421749

[B39] Lee H. (2011). Inequality as an explanation for obesity in the United States. Sociol. Comp. 5, 215–232. 10.1111/j.1751-9020.2010.00355.x

[B40] Lerner M. J. (1965). Evaluation of performance as a function of performer's reward and attractiveness. J. Pers. Soc. Psychol. 1, 355–360. 10.1037/h0021806 14328745

[B41] Lerner M. J. Miller D. T. (1978). Just world research and the attribution process: Looking back and ahead. Psychol. Bull. 85, 1030–1051. 10.1037/0033-2909.85.5.1030

[B42] Loewenstein G. (2018). Self-control and its discontents: a commentary on Duckworth, Milkman, and Laibson. Psychol. Sci. Public Int. 19, 95–101. 10.1177/1529100619828401 30760174

[B43] Logel C. Stinson D. A. Brochu P. M. (2015). Weight loss is not the answer: a well-being solution to the “obesity problem”. Soc. Personal. Psychol. Compass 9, 678–695. 10.1111/spc3.12223

[B44] Madan S. Nanakdewa K. Savani K. Markus H. R. (2020). The paradoxical consequences of choice: often good for the individual, perhaps less so for society? Curr. Dir. Psychol. Sci. 29, 80–85. 10.1177/0963721419885988

[B45] McGuire J. T. Kable J. W. (2013). Rational temporal predictions can underlie apparent failures to delay gratification. Psychol. Rev. 120:395. 10.1037/a0031910 23458085 PMC3773987

[B46] Milyavskaya M. Saunders B. Inzlicht M. (2021). Self-control in daily life: prevalence and effectiveness of diverse self-control strategies. J. Pers. 89, 634–651. 10.1111/jopy.12604 33128774

[B47] Mooijman M. Meindl P. Oyserman D. Monterosso J. Dehghani M. Doris J. M.. (2018). Resisting temptation for the good of the group: binding moral values and the moralization of self-control. J. Pers. Soc. Psychol. 115:585. 10.1037/pspp0000149 28604018

[B48] Mukhopadhyay A. Johar G. V. (2005). Where there is a will, is there a way? Effects of lay theories of self-control on setting and keeping resolutions. J. Cons. Res. 31, 779–786. 10.1086/426611

[B49] Newman G. E. De Freitas J. Knobe J. (2015). Beliefs about the true self explain asymmetries based on moral judgment. Cogn. Sci. 39, 96–125. 10.1111/cogs.12134 25039306

[B50] Pancrazi R. van Rens T. Vukotic M. (2022). How distorted food prices discourage a healthy diet. Sci. Adv. 8:eabi8807. 10.1126/sciadv.abi8807 35353561 PMC11093214

[B51] Papadopoulos S. Brennan L. (2015). Correlates of weight stigma in adults with overweight and obesity: a systematic literature review. Obesity 23, 1743–1760. 10.1002/oby.21187 26260279

[B52] Pereira-Miranda E. Costa P. R. Queiroz V. A. Pereira-Santos M. Santana M. L. (2017). Overweight and obesity associated with higher depression prevalence in adults: a systematic review and meta-analysis. J. Am. Coll. Nutr. 36, 223–233. 10.1080/07315724.2016.1261053 28394727

[B53] Pigeyre M. Rousseaux J. Trouiller P. Dumont J. Goumidi L. Bonte D.. (2016). How obesity relates to socio-economic status: identification of eating behavior mediators. Int. J. Obes. 40, 1794–1801. 10.1038/ijo.2016.109 27377952

[B54] Rafieian H. Sharif M. A. (2023). It's the effort that counts: the effect of self-control on goal progress perceptions. J. Market. Res. 60, 527–542. 10.1177/00222437221123969

[B55] Rawn C. D. Vohs K. D. (2011). People use self-control to risk personal harm: an intra-interpersonal dilemma. Person. Soc. Psychol. Rev. 15, 267–289. 10.1177/1088868310381084 20807858

[B56] Reynolds J. J. McCrea S. M. (2019). Environmental constraints on the functionality of inhibitory self-control: sometimes you should eat the donut. Self Ident. 18, 60–86. 10.1080/15298868.2017.1354066

[B57] Righetti F. Finkenauer C. (2011). If you are able to control yourself, I will trust you: the role of perceived self-control in interpersonal trust. J. Pers. Soc. Psychol. 100:874. 10.1037/a0021827 21319912

[B58] Savani K. Rattan A. (2012). A choice mind-set increases the acceptance and maintenance of wealth inequality. Psychol. Sci. 23, 796–804. 10.1177/0956797611434540 22700330

[B59] Savani K. Stephens N. M. Markus H. R. (2011). The unanticipated interpersonal and societal consequences of choice: victim blaming and reduced support for the public good. Psychol. Sci. 22, 795–802. 10.1177/0956797611407928 21537057

[B60] Silveira E. A. Mendonça C. R. Delpino F. M. Souza G. V. E. de Souza Rosa L. P. de Oliveira C.. (2022). Sedentary behavior, physical inactivity, abdominal obesity and obesity in adults and older adults: a systematic review and meta-analysis. Clin. Nutr. ESPEN 50, 63–73. 10.1016/j.clnesp.2022.06.001 35871953

[B61] Sunstein C. R. (2016). Fifty shades of manipulation. J. Market. Behav. 1, 213–244. 10.1017/CBO9781316493021.005

[B62] Sutin A. R. Stephan Y. Terracciano A. (2015). Weight discrimination and risk of mortality. Psychol. Sci. 26, 1803–1811. 10.1177/0956797615601103 26420442 PMC4636946

[B63] Swinburn B. A. Sacks G. Hall K. D. McPherson K. Finegood D. T. Moodie M. L.. (2011). The global obesity pandemic: shaped by global drivers and local environments. Lancet 378, 804–814. 10.1016/S0140-6736(11)60813-1 21872749

[B64] Turel O. He Q. Xue G. Xiao L. Bechara A. (2014). Examination of neural systems sub-serving Facebook “addiction”. Psychol. Rep. 115, 675–695. 10.2466/18.PR0.115c31z8 25489985

[B65] Twenge J. M. Martin G. N. Spitzberg B. H. (2019). Trends in US Adolescents' media use, 1976–2016: the rise of digital media, the decline of TV, and the (near) demise of print. Psychol. Pop. Media Cult. 8, 329–345. 10.1037/ppm0000203

[B66] Westbury S. Oyebode O. Van Rens T. Barber T. M. (2023). Obesity stigma: causes, consequences, and potential solutions. Curr. Obes. Rep. 12, 10–23. 10.1007/s13679-023-00495-3 36781624 PMC9985585

[B67] Yin Y. Savani K. Smith P. K. (2022). Power increases perceptions of others' choices, leading people to blame others more. Soc. Psychol. Personal. Sci. 13, 170–177. 10.1177/19485506211016140

[B68] Zuboff S. (2019). The Age of Surveillance Capitalism: The Fight for a Human Future at the New Frontier of Power. New York, NY: Public Affairs.

